# Frequency of mutations in BRAF, NRAS, and KIT in different populations and histological subtypes of melanoma: a systemic review

**DOI:** 10.1097/CMR.0000000000000628

**Published:** 2019-07-03

**Authors:** Luz Dary Gutiérrez-Castañeda, John A. Nova, José D. Tovar-Parra

**Affiliations:** Teaching and Research Office, Hospital Universitario—Centro Dermatológico Federico Lleras Acosta, E.S.E., Bogotá, Colombia

**Keywords:** BRAF, KIT, Melanoma, meta-analysis, mutation, NRAS

## Abstract

The presence of mutations of *BRAF*, *NRAS*, and *KIT* genes is recognized as playing a role during carcinogenesis. Our study aims to evaluate and review other studies that present the frequency of mutations of *BRAF*, *NRAS*, and *KIT* genes for different populations, and analyse correlation to their clinical-pathological characteristics and to the demographics of melanoma. Thirty-two articles were selected from a collection of published literature studying 6299 patients. The parameters for correlation to different variables were calculated by odds ratio, for random and single effects. 38.5% of patients present *BRAF* gene mutations, 16.4% in *NRAS*, and 10% in *KIT*. Mutations of the *BRAF* gene were correlated to superficial spreading melanoma (odds ratio = 1.31), localization in the torso (odds ratio = 1.42) and presence of metastases. Mutations in *NRAS* were correlated to nodular melanoma (odds ratio = 1.57), localized in the limbs (odds ratio = 1.31). Mutations of the *KIT* gene were correlated to mucosal melanoma (odds ratio = 1.59). Populations in Brazil, the US, Sweden, Italian, and Australia were found to be correlated to mutations of BRAF and melanoma. Populations in Italy, Sweden, Spain, and the US were found to be correlated to mutations of NRAS. Populations in Japan, China, Turkey, Canada, and Russia were found to be correlated to mutations of KIT. Data correlated to the presence of melanoma and population type is due to the amount of studies performed across of globe.

## Abstract 2

Supplemental Digital Content is available in the text.

## Introduction

In the last few years there has been an increase of melanoma incidence in all populations [1]. Presently, this represents 5.3% of all skin cancers, and approximately 1.5% (9320) of patients die from this disease [2]. In 2020 it is projected that 279 938 new cases will be diagnosed, and it is estimated that around 67 809 will die as a result [3].

The incidence rate of cutaneous melanoma is greater in white population groups compared to Hispanic, African-American, Indo-American, and Asian population groups [4]. The risk of melanoma increases with age and has been described as having a 1.5 incidence rate greater for males than for females [5]. Other risk factors include exposure to ultraviolet radiation, the presence of many nevus, skin colour, hair colour, eye colour, altitude, and latitude. All these factors contribute to a change in the molecular machinery of melanocytes, which transforms a normal cell into a tumourous cell [6].

Signalling pathways altered during carcinogenesis of melanoma include the protein kinase activated by mitogen (MAPK), phosphoinositide 3-kinase (PI3K), tumour suppressor retinoblastoma, and p53 protein pathways [7]. Pathways act either collectively or individually to regulate growth, proliferation, and apoptosis. Prolonged activation of these pathways relates to the proliferation, invasion, metastasis, and survival of melanomas [7].

Mutations of *BRAF*, *NRAS*, and *KIT* genes have been correlated to the development of melanoma. These mutations have been described as being mutually excluding [8,9]. The tyrosine-protein kinase Kit acts as a cell surface-cells receptor, participates in the normal growth of melanocytes and intervenes in the activation of MAPK/PI3K pathways [8]. *KIT* gene mutations are present in 39% of mucosal melanomas (MM), 36% of acral lentiginous melanomas (ALM), and in 28% of skin with chronic solar damage. The majority of reported mutations are found in exons 9, 11, 13, and 17, and represent between 5 and 10% of mutations of diagnosed melanomas [10,11].

NRAS oncogene is a member of the superfamily of p21 GTPase proteins. These proteins cause intrinsic GTPase activity, functioning as a molecular switch for transmission in the regulator signals. They participate in the activation of MAPK/PI3K pathway, during cellular proliferation and survival [1,12]. The most common NRAS gene mutations are Q61R and Q61K and represent around 25% of mutations for biopsies of patients with melanoma [12].

Another gene implicated in the pathogenesis of melanoma is oncogene *BRAF*. This gene encodes a serine-threonine protein kinase of the RAF (rapidly accelerated fibrosarcoma) family. This protein plays an important role in the regulation of MAPK pathways signalling, causing changes in cell cycle, differentiation, and apoptosis [13]. The frequencies of BRAF mutation ranged from 20 to 80% [14]. The most common BRAF alteration detected in human cancers includes the V600E mutation, which is located in the activation segment of the kinase. This mutation represents between 60 and 80% of all mutations for this gene [15].

Regardless of the conclusions stated previously, it is not known whether the frequency and correlations to mutations of these genes and melanoma are the same for all populations, or if differences exist for some of the populations analysed. Additionally, how the influence of ethnic variability affects histological and clinical presentation for tumours is not known. For this reason, the aim of revising all studies evaluating the frequency of mutations of *BRAF*, *NRAS*, and *KIT* genes are to determine the correlation of the clinical-pathological characteristics of melanomas, and the demographic characteristics of the analysed populations.

## Materials and methods

### Criteria for eligibility and data recollection

The following databases were consulted: Medline, accessed through PubMed (https://www.ncbi.nlm.nih.gov/pubmed/), Science Direct (http://www.sciencedirect.com/), and to consult literature from Latin America Scielo (http://www.scielo.cl/) was accessed. The strategy used to search for data was a combination of the words ‘melanoma AND mutations OR mutation OR mutat* AND BRAF OR B-RAF AND NRAS OR N-RAS AND C-KIT OR KIT’, ‘melanoma AND mutations OR mutation OR mutat* AND BRAF OR B-RAF’, ‘melanoma AND mutations OR mutation OR mutat* AND NRAS OR N-RAS’, ‘melanoma AND mutations OR mutation OR mutat* AND C-KIT OR KIT’. Studies included those published between January 1st 2005 and November 30th 2017, both in English and Spanish, as well as observational studies (Supplemental digital content 1, http://links.lww.com/MR/A157 lists the strategy for all queries in database search).

### Exclusion and inclusion criteria

The studies revised met the following criteria: (1) Studies published between 2005 and 2017. (2) Studies written in English or Spanish. (3) Studies with the frequency of *BRAF* or *B-RAF*, *NRAS* or *N-RAS* and *KIT* or *C-KIT* gene mutations. (4) Studies reporting types and subtypes of melanoma. (5) Studies describing the population where mutations were analysed.

**Fig. 1:**
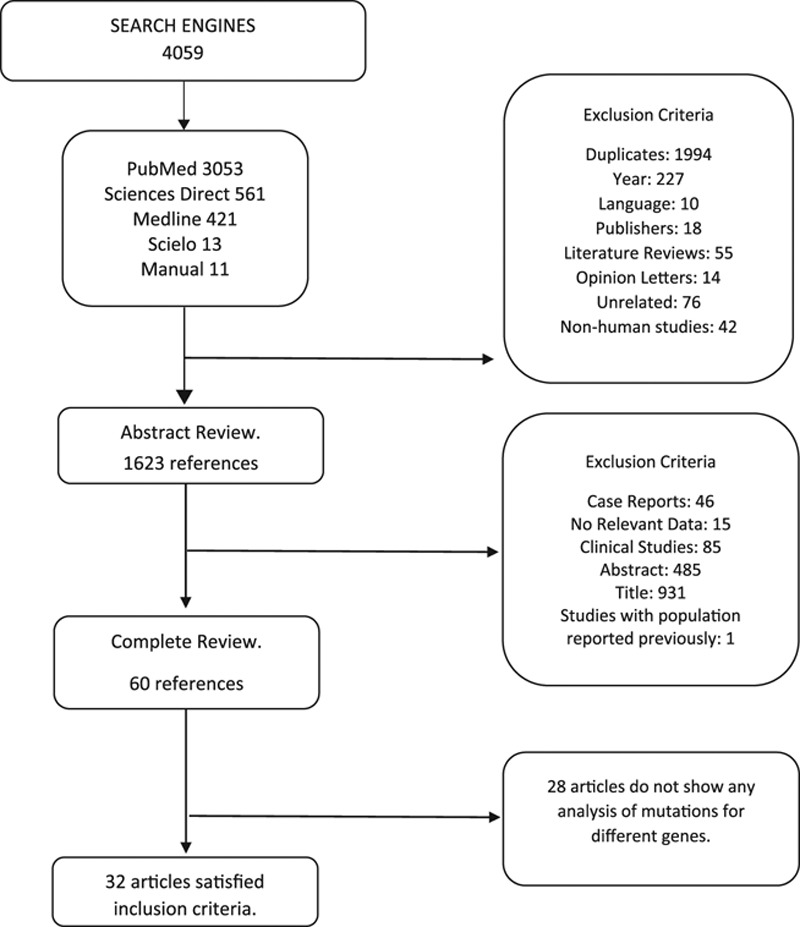
Flow diagram illustrating how studies were selected, inclusion and exclusion criteria.

The exclusion criteria were: publication before 2005, duplicate articles, case reports, languages other than Spanish and English, letters to the editor, reviews, studies in cell lines, studies that did not include clinical data, or did not report the location of the analysed population, abstracts, clinical studies or clinical trials and titles. Additionally, studies that reported double findings for the same population were excluded according to the hospital or reference center, date of publication, group of authors, country and bibliographical reference, or for data published previously (Fig. 1).

### Data extraction

Two reviewers independently evaluated published literature in order to preserve homogeneity and to eliminate subjectivity for the extraction of data. In the case of disagreement, the issue was resolved through a group discussion involving both reviewers. From each selected study the following information was obtained: name of the article, original author, year of publication, melanoma subtype, number of patients, country of origin of analysed population, molecular analysis and sequencing method, anatomical location of melanoma, mutations in *BRAF*, *NRAS*, and *KIT* gene, sex, age, chronic solar exposure, metastasis, and ulceration.

### Statistical analysis

Multiple regressions were used to review the influence of the variables extracted from each one of these studies. A review of the correlation to mutations and the place of origin of the patient was proposed using the odds ratio (OR) with a confidence interval (CI) of 95%. These calculations were performed using Open Meta-Analyst software free version. For fixed effects, the Mantel–Haenszel method was used. The choice of the method of analysis was taken according to Q value in order to observe the heterogeneity between analysed studies, following the chi-square distribution with k − 1 degrees of freedom for homogenous studies. The inconsistency of the studies was quantified with the I^2^ statistic, that allowed us to obtain a total estimation of the variability or inconsistency of analysed studies and the value that oscillates between 0 and 100%. In terms of heterogeneity, the meta-analysis was performed using the Binary Random-Effects model, and the Random-Effects (DerSimonian-Laird) method of meta-analysis for random effects. In order to determine the bias for publication related to negative results, the *comprehensive meta-analysis* V.13 (free version) software was used since they are cited less frequently, thus, making them scarcer in database searches. A funnel plot analysis was performed and presented asymmetry parameter values of *P* = <0.05, which indicated presence of bias.

## Results

From the review of the database search, a total of 4059 articles were included. Once verified, 61 articles were considered relevant and were reviewed in their entirety. Thirty-two articles, studying 6299 patients, satisfied the inclusion criteria for clinical-pathological and demographic variable analysis (Table 1). Of the 32 articles, 26 reported mutations of the *BRAF* gene, 17 of the *NRAS* gene, and 13 of the *KIT* gene. The number of patients were 5480, 3904, and 1437 for analysis of mutations of *BRAF*, *NRAS*, and *KIT* genes, respectively. The frequency of *BRAF* mutations gene was 38.5% (2113), 16.4% (641) for *NRAS*, and 10% (150) for *KIT*.

**Table 1:**
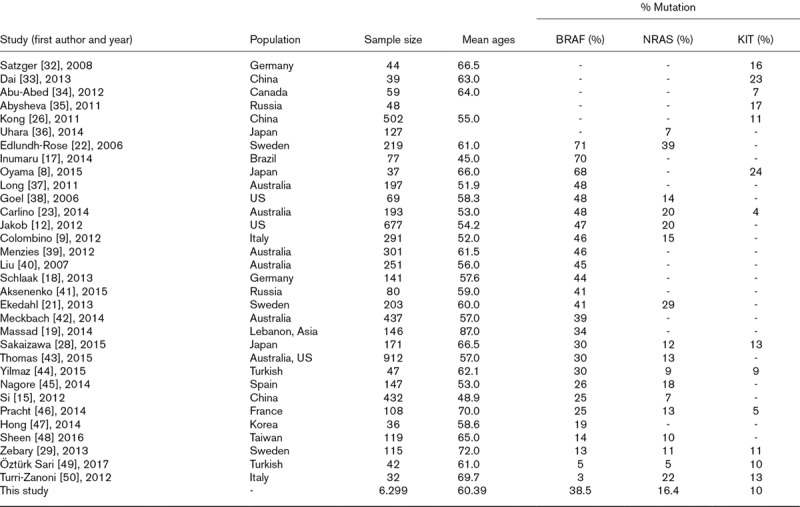
Frequency of mutations of BRAF, NRAS, and KIT genes for studies analysed, sample size, and analysed population type

For frequency of mutations of the *BRAF* gene, 27 articles were reported according to sex, 15 for *NRAS* gene, and 8 for *KIT* gene. 43.6, 39, and 56% of patients who tested positive for *BRAF*, *NRAS*, and *KIT* gene are female, respectively. Regarding mutations in males and females for these genes, there was no correlation found between them.

**Fig. 2:**
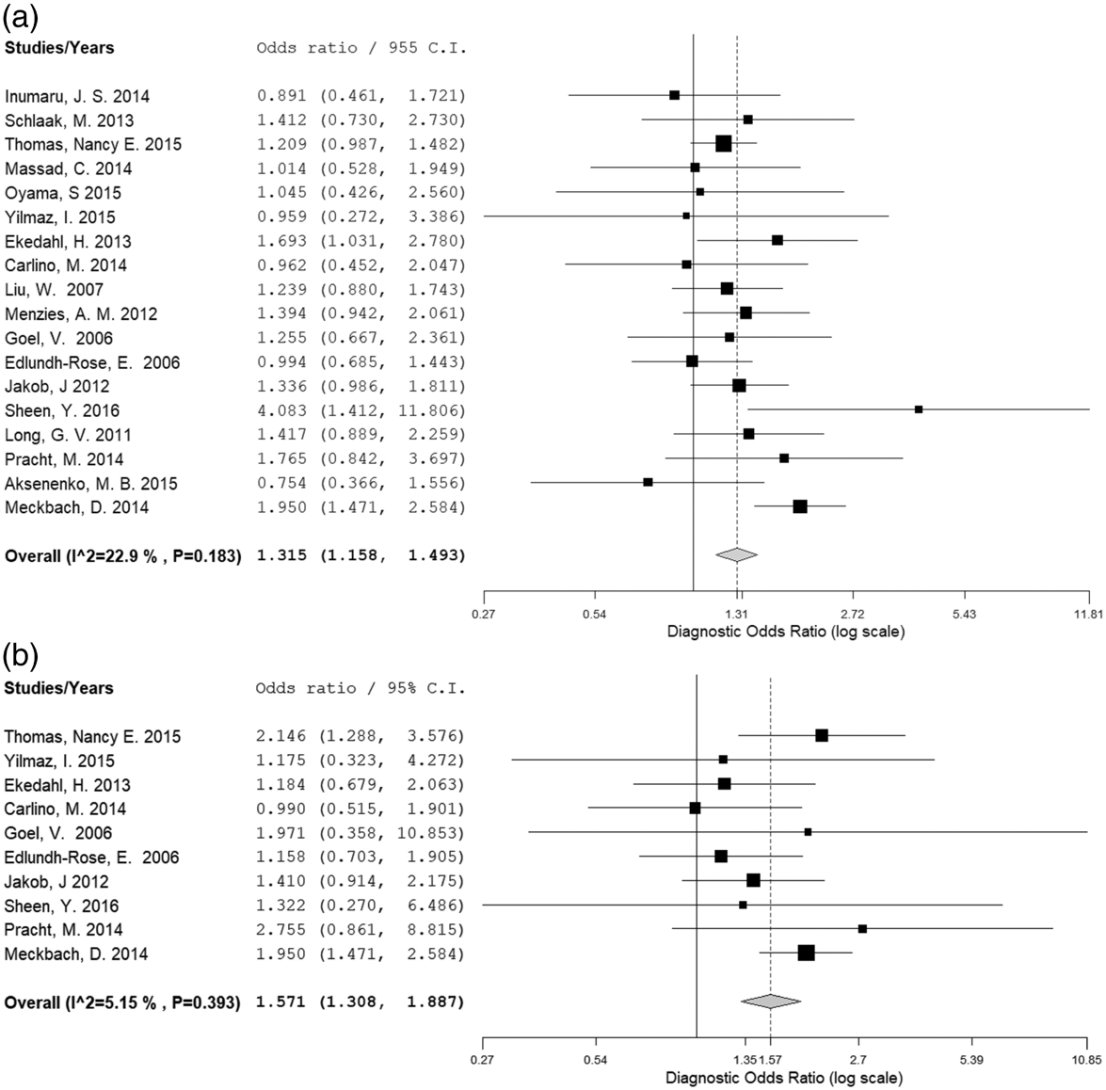
(a) Correlation to *BRAF* gene mutations and superficial spreading melanoma with an odds ratio value corresponding to a CI of 95% for studies performed. (b) Odds ratio with a CI of 95% for correlation to NRAS mutations and nodular melanoma. CI, confidence interval.

A subsequent analysis was performed for the mutations reported of the *BRAF* gene and their relation to the histological subtype, for 25 of the 32 studies included for review. Of the 2075 patients who tested positive for BRAF mutation, 886 (42.7%) presented superficial spreading melanoma (SSM) histological subtype (OR = 1. 31, 95% CI 1.15–1.49, *P* < 0.001) (Fig. 2a). On the contrary, for nodular melanomas (NM), lentigo malignant melanoma (LMM), ALM, uveal melanoma (UM), MM, primary melanoma, and undifferentiated melanoma there was no relation found to *BRAF* gene mutations and histological subtypes.

An analysis of mutations in the *NRAS* gene and the histological characteristics of melanoma were reported in 17 articles. Of the 641 patients who presented mutations in *NRAS* gene, 21.1% (135) presented NM, and 19.1% (127) presented the histological subtype of undifferentiated melanoma. A statistical analysis found a correlation to mutations of *NRAS* gene and nodular subtype (OR = 1.57, 95% CI 1.30–1.88, *P* < 0.001) (Fig. 2b), and with undifferentiated (OR = 1.35, 95% CI 1.06–1.71). An analysis of the mutations of the *KIT* gene and their relation to different melanoma subtypes was not found to be correlated to histological subtypes. With ALM, an analysis of fixed effects was performed according to the homogeneity of collected data (OR = 1.45, 95% CI 0.89–2.11, Q = 7.97, df = 6, *P* = 0.240, I^2^ = 24.74).

**Fig. 3:**
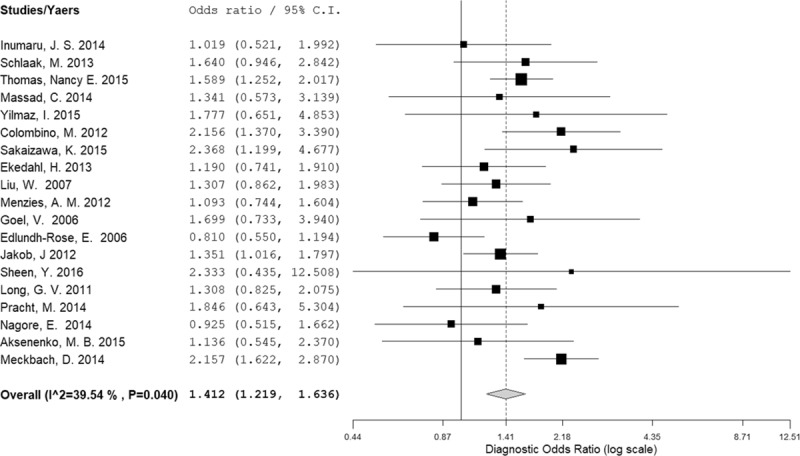
Correlation to BRAF mutations and anatomical localization of melanoma (torso).

The correlation of mutations and anatomical location of melanoma was also analysed. Thirty-four percent (674/1978) of patients with melanoma were reported to have presented a correlation to melanomas located in the torso with mutation of the BRAF gene (OR = 1.41, 95% CI 1.21–1.63, *P*< 0.001) (Fig. 3). The correlation between mutations of the *NRAS* gene and the location of melanoma in the limbs was significant. 34.7% of patients (212/610) had mutations in limbs (OR = 1.31, 95% CI 1.01–1.70, *P* = 0,036). For anatomical locations in head, neck, torso, mucosa, feet, and hands no correlation was found for mutations of *NRAS* gene.

Thirteen of all studies analysed reported the melanoma localization and the presence of *KIT* gene mutations. A correlation was shown for localization in mucosa and presence of *KIT* gene mutations (OR = 1.59, 95% CI 1.10–2.31, *P* < 0.013, Q = 12.4, df = 8).

Five of the 26 studies, that analysed *BRAF* gene mutations report the relation to metastatic sites. The presence of mutations in *BRAF* gene, and their relationship to metastases is significant (OR = 1.95, 95% CI 1.13–3.35, Q = 18.8, df = 5, *P* = 0.016 and I^2^ = 78), but for mutations on *NRAS* and *KIT* gene this relationship was not found.

### Correlated risk factors

There was no correlation found to chronic solar exposure and presence of mutations in BRAF (OR = 0.941, 95% CI 0.729–1.21, Q = 8.391, df = 9, *P* = 0.495 and I^2^ = 0), or NRAS (OR = 0.362, 95% CI 0.06–1.97, *P* = 0.24), and KIT (OR = 1.00, 95% CI 0.323–3.12, *P* = 0.99). A correlation to ulceration presence and *NRAS* gene mutations was found (OR = 2.90, 95% CI 1.24–6.73, *P* = 0.003). Such a factor is not found to be correlated to the presence of *BRAF* gene mutations, nor for *KIT* gene mutations.

### Patient origin country

One aspect that has not been found to be related to the presence of mutations in the population’s origin country. According to this statement, an analysis was performed to determine if a relationship exists between mutations of *BRAF*, *NRAS*, and *KIT* genes, and population type according to country of origin. No global relationship was found for the studied populations and the presence of BRAF mutations (OR = 1.04, 95% CI 0.99–1.10, *P* < 0.090). Additionally, a significant statistic was found for the heterogeneity of the collected data in the analysed articles (Q = 209.42, df = 12, *P* < 0.001). Given the observation of bias, an independent analysis was performed for each population.

The correlation to population and the presence of *BRAF* gene mutations was found by independent analysis; in Brazil (OR = 2.91, 95% CI 1.47–2.97), Australia (OR = 1.13, 95% CI 1.02–1.25), Italy (OR = 1.25, 95% CI 1.02–1.54), US (OR = 1.33, 95% CI 1.18–1.49), and Sweden (OR = 1.41, 95% CI 1.20–1.65). Populations analysed from other sources did not demonstrate such correlations.

**Fig. 4:**
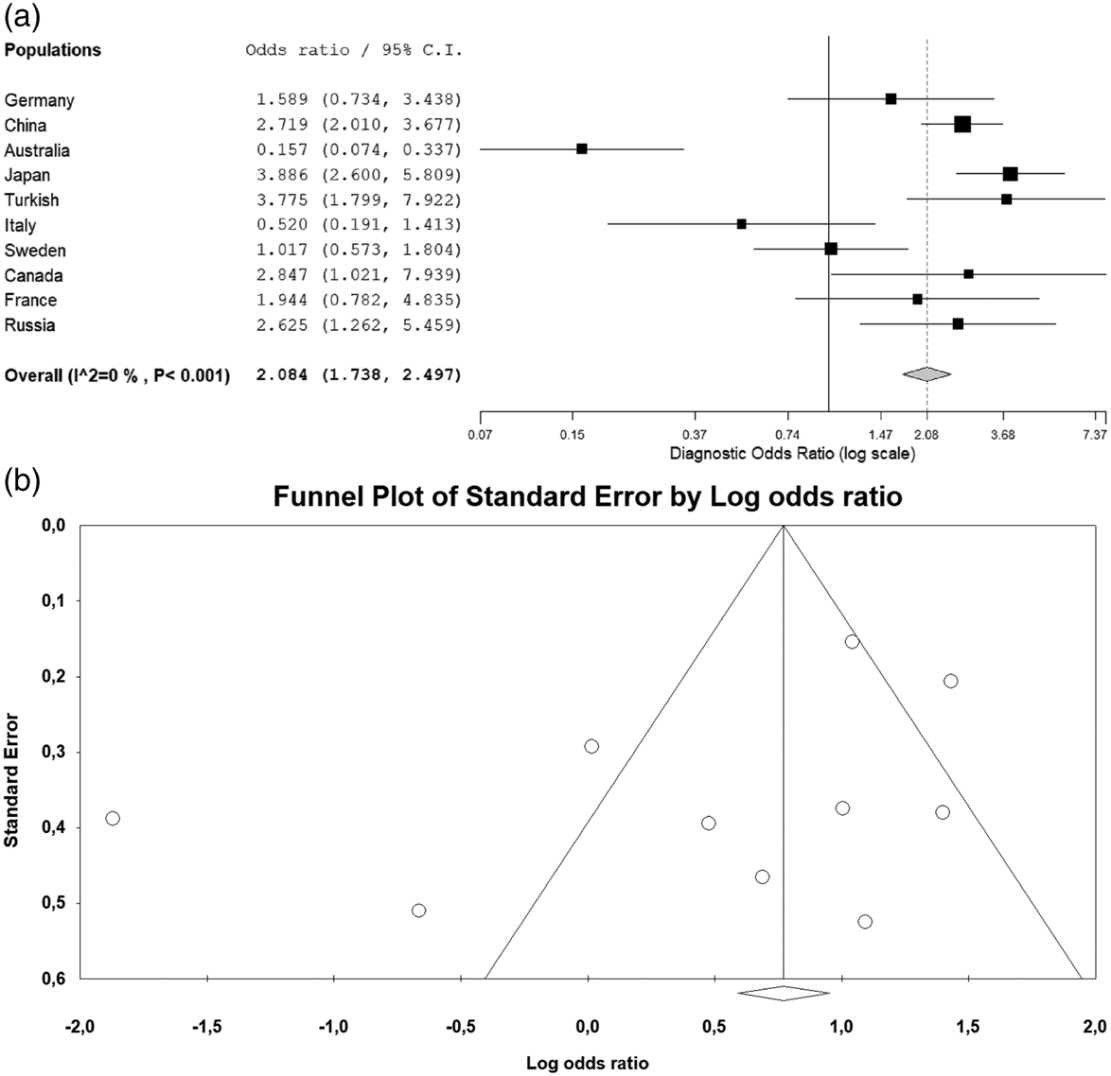
(a) Correlation to *KIT* gene mutations and population type. (b) Funnel plot of *KIT* gene mutations and population type.

The global relation between the analysed population and the presence of mutations in *NRAS* gene was shown to be correlated (OR = 1.34, 95% CI 1.23–1.47, *P* < 0.001). The analysis for the presence of mutations of this gene, and the specific population, was shown to be significant for Italy (OR = 1.52, 95% CI 1.11–2.07), Sweden (OR = 2.87, 95% CI 2.36–3.49), Spain (OR = 1.80, 95% CI 1.18–2.74), and the US (OR = 1.89, 95% CI 1.61–2.23). The analysis of this relation to the global population and the presence of mutations in *KIT* gene was shown to be correlated as well (OR = 2.08, 95% CI 1. 73–2.49, *P* < 0.001). The populations in which the greatest correlation was found are China (OR = 2.71, 95% CI 2.01–3.67), Japan (OR = 3.88, 95% CI 2.60–5.80), Turkey (OR = 3.77, 95% CI 1.79–7.92), Canada (OR = 2.84, 95% CI 1.02–7.93), and Russia (OR = 2.62, 95% CI 1.26–5.45) (Fig. 4a).

Funnel plot analysis demonstrates no bias in published studies which analyse mutations in *BRAF*, *NRAS*, and *KIT* genes for patients with melanoma, according to their sex, melanoma subtype, localization of melanoma, chronic solar exposure, and ulceration. Nevertheless, a published bias is present in the analysis of genes, the presence of metastasis, and population type (Fig. 4b).

## Discussion

Melanoma has been understood according to the identification of mutations and genetic changes, thus, allowing for the development of a more effective approach to the disease, especially in advanced cases. The knowledge of the frequencies of mutations in the *BRAF*, *KIT*, and *NRAS* genes seen along the study in the different geographical regions with the pathological and clinical characteristics, is important for treatment planning and the sifting of evidence.

For the present meta-analysis, correlations to patients with BRAF, NRAS, and KIT mutations, clinical-pathological characteristics and risk factors correlated to the development of melanoma were grouped together. Such information may help to guide development of therapeutic strategies and also, aid in the identification of specific risk factors for each population.

Various population studies demonstrate correlation to clinical-pathological characteristics, histological subtypes, and presentation of *BRAF* gene mutations; nevertheless, these results are not conclusive and vary according to the population and samples analysed. Different studies have reported that BRAF mutations are more frequent in skin that has been exposed to sunlight intermittently, which is correlated to a melanoma subtype with surface spreading and localization in the torso [16]; nonetheless, we do not find any correlation between the presence of BRAF mutations and solar exposure. Likewise, some studies performed on populations from Italy (Libra *et al.* [13]) and Brazil (Inumaru *et al.* [17]) found that the presence of the V600 mutation, of *BRAF* gene, is not correlated to factors such as sex, melanoma type, chronic solar exposure, presence of metastases, and ulceration. This differs from a study performed on a German population where a correlation to the presence of BRAF mutations and localization in the torso was found (<0.0001) [18]. Another study of 146 patients in Lebanon (Asia) also reported the presence of subcutaneous metastatic melanoma correlated to the presence BRAF mutations [19]. In this meta-analysis, we found a correlation to the presence of BRAF mutations for the histological subtype of SSM with localization in the torso and the presence of metastases, but not for solar exposure.

*BRAF* gene mutations are considered as driver mutations due to their presence in nevus, correlated to the development of melanoma and PMs and metastasic outgrowths [20]. In the present meta-analysis, a correlation to the presence of BRAF mutations and the presence of metastatic melanomas was found. Edlundh-Rose *et al*. and Ekedahl *et al.* found the presence of mutations for this gene in PM and metastases in samples from such patients [21,22]. Conversely, Carlino *et al*. [23] reported no correlation to the presence of *BRAF* and *NRAS* gene mutations and the progression of metastatic disease.

The presence of mutations of NRAS, and *KIT* genes has also been correlated to the subtype and localization of melanoma. Devitt *et al.* in Australian patients, found a correlation to NRAS mutations and the histological subtype of nodular melanoma (*P* = <0.001) [24]. Similarly, Jakob *et al*. [12] in 2012 studied 677 samples of the American population and reported a correlation to anatomical localization (*P* = <0.001) and to subtypes with a greater degree of correlation to PM. As would be expected, the data analysed in this study demonstrated a correlation to the presence of mutations and localization of melanoma in lower and upper limbs and NM subtype.

The mutations of the *KIT* gene are found with greater frequency for mucosal melanoma (30%), unlike for *BRAF* and *NRAS* genes. The mutations of this gene are heterogeneous and are found along the length of exon 11. Additionally, these mutations occur independently from mutations of *BRAF* and *NRAS* genes, indicating that the pathogenesis of cutaneous melanoma and mucosal melanoma occurs differently [25].

In cutaneous melanoma, the mutation of KIT has been found more frequently for ALM [26]. This melanoma subtype is prevalent for Asian populations and in Latin America. Despite this, we have not found studies in Latin American populations that allow us to verify the correlation between KIT and ALM mutations. Furthermore, KIT mutation has been related to chronic solar damage [27], and yet our study could not affirm said correlation (OR = 1.0, CI 0.3–3.1); such a result is possible due to population diversity and the frequency of histological subtype in the studies analysed. According to what was reported by Sakaizawa *et al.* [28] in Japanese patients where the correlation to melanoma type and anatomical localization was analysed (*P* = 0.554). Correspondingly, Zebary *et al*. [29] in 2013 did not observe any correlation between melanoma type, ulceration, clinical and histopathological characteristics for patients analysed with ALM (*P* = 0.039) for KIT mutations. In this meta-analysis, the correlation to clinical-pathological characteristics, for patients with melanoma diagnosis, was shown.

As was previously stated, several studies have identified the correlation to *BRAF*, *NRAS*, and *KIT* gene mutations, for different clinical-pathological characteristics and clinical risk factors. In this meta-analysis, such an existing correlation is confirmed independently of the period of time that has been measured. Although the incidence of melanoma continues to increase heterogeneously across the globe [30]. For example, Lee *et al*. [31], performed a meta-analysis in 2010, including 36 published articles between 1989 and 2010 where a correlation was conclusively found to SSM and NM and the presence of BRAF and N-RAS mutations. We found this correlation in published studies between 2005 and 2017. Additionally, we found a correlation to ulceration and the presence of mutations in NRAS.

On the other hand, for isolated populations various studies demonstrate the analysis of mutations and their correlation to melanoma. Consequently, it has not demonstrated whether the correlation to *BRAF*, *NRAS*, and *KIT* mutations gene is related to population type, as well as for the geographical characteristics of the region where the population resides. The meta-analysis presented a correlation to the presence of *BRAF* gene mutations for patients with melanoma and the populations of Brazil, Australia, Italy, US, and Sweden.

Hence, the presence of mutations of the *NRAS* gene and populations from Italy, Sweden, Spain, and the US is found to be correlated. This coincides with the greater frequency of melanoma subtype of SSM and NM for these populations and with the frequency of mutations for these two genes and subtypes, compared to the MM and ALM. However, a correlation is found for different geographical regions when the presence of *KIT* gene mutations are analysed. The greatest correlations were found in the populations of Japan followed by Turkey, Canada, China, and Russia, respectively. This indicates that it is possible that a correlation found by this study, for the region analysed, is due to the greater frequency of mutations for histological subtypes. These findings allow us to generate strategies for prevention and treatment, based in a molecular point of view, for populations that are at risk of developing melanoma.

The difference in the frequency of mutations of *BRAF*, *NRAS*, and *KIT* genes for different populations reflects existing variability. It may be more adequate, in order to homogenize the study of melanoma, to collect information on a region by region basis. In this study, correlation is only analysed for populations from certain countries and melanoma subtypes when such mutations are found.

Our study has limitations. This is a systematic review and meta-analysis of the literature of reports for frequency mutations of *BRAF*, *NRAS*, and *KIT* genes around the world and is more likely to be compromised by selection and enrichment bias of patients from a specific region of a country; it being the case that patient selection in the majority of studies is performed at reference centers. The combined estimate may be overestimating the frequency of mutations and therefore, may not be representing differences for separate regions in one country. Additionally, the analysis of the frequency of mutations and their correlation to the analysed population represents published bias due to the variability of incidence for disease. The majority of studies performed occur in White populations with high rates of incidence for some melanoma subtypes.

## Acknowledgements

We acknowledge the Hospital Universitario–Centro Dermatológico Federico Lleras Acosta, E.S.E.

This study was supported by the Hospital Universitario—Centro Dermatológico Federico Lleras Acosta, E.S.E.

## Conflicts of interest

There are no conflicts of interest.

## Supplementary Material
