# Supplementary material for: Frequency of mutations in BRAF, NRAS, and KIT in different populations and histological subtypes of melanoma: a systemic review
Source: Melanoma Res. 2019 Jul 3;30(1):62–70. doi: 10.1097/CMR.0000000000000628 (PMC6940026; doi:10.1097/CMR.0000000000000628)
Supplement: Supplementary file 1 [file mr-30-62-s001.pdf]

Supplement 1. Strategy for database search

Pubmed

- (melanoma [MeSH Terms]AND ((NRAS[Title/Abstract]) OR (N-RAS[Title/Abstract])) AND ((BRAF [Title/Abstract]) OR (B-RAF [Title/Abstract])) AND ((Mutation [Text Word]) OR Mutatio*[Text Word]) OR (OR mutat**[Text Word])) AND ((C-KIT[Title/Abstract]) OR KIT[Title/Abstract]))
- (melanoma [MeSH Terms]) AND ((Mutation [Text Word]) OR Mutatio*[Text Word]) OR (OR mutat**[Text Word])) AND ((NRAS[Title/Abstract]) OR N-RAS[Title/Abstract])).
- (melanoma [MeSH Terms]) AND ((BRAF [Title/Abstract]) OR (B-RAF [Title/Abstract])) AND ((Mutation [Text Word]) OR Mutatio*[Text Word]) OR (OR mutat**[Text Word]))
- (melanoma [MeSH Terms] AND ((C-KIT[Title/Abstract]) OR KIT[Title/Abstract])) AND ((Mutation [Text Word]) OR Mutatio*[Text Word]) OR (OR mutat**[Text Word])).

Science direct

- Melanoma AND Mutation (B-RAF OR BRAF) AND (NRAS or N-RAS) AND (C-KIT OR KIT)

MEDLINE

- Melanoma AND mutation AND (B-RAF OR BRAF) AND (NRAS or N-RAS) AND (C-KIT OR KIT), Melanoma AND mutation AND BRAF OR B-RAF, Melanoma AND mutation AND NRAS OR N-RA, Melanoma AND mutation AND KIT OR C-KIT

Google scholar (alert service)

- Melanoma|BRAF|B-RAF|NRAS|N-RAS|KIT|C-KIT|mutation|mutat

Scielo

- Melanoma AND BRAF OR B-RAF
- Melanoma AND NRAS OR N-RAS
- Melanoma AND KIT OR c-KIT
- Melanoma
